# From graphene oxide towards aminated graphene: facile synthesis, its structure and electronic properties

**DOI:** 10.1038/s41598-020-63935-3

**Published:** 2020-04-23

**Authors:** Maxim K. Rabchinskii, Sergei A. Ryzhkov, Demid A. Kirilenko, Nikolay V. Ulin, Marina V. Baidakova, Vladimir V. Shnitov, Sergei I. Pavlov, Ratibor G. Chumakov, Dina Yu. Stolyarova, Nadezhda A. Besedina, Aleksandr V. Shvidchenko, Dmitrii V. Potorochin, Friedrich Roth, Dmitry A. Smirnov, Maksim V. Gudkov, Maria Brzhezinskaya, Oleg I. Lebedev, Valery P. Melnikov, Pavel N. Brunkov

**Affiliations:** 1 Ioffe Institute, 26 Politekhnicheskaya, 194021 Saint Petersburg, Russia; 2 ITMO University, 49 Kronverksky Pr., 197101 Saint Petersburg, Russia; 3 NRC “Kurchatov Institute”, 1 Akademika Kurchatova pl., 123182 Moscow, Russia; 4 St. Petersburg Academic University, Khlopin St. 8/3, 194021 Saint Petersburg, Russia; 5 Semenov Institute of Chemical Physics of Russian Academy of Sciences, Kosygina St., 4, 119991 Moscow, Russia; 6 Technische Universität Bergakademie Freiberg, Akademiestraße 6, 09599 Freiberg, Germany; 7 Deutsches Elektronen-Synchrotron DESY, 85 Notkestraße, Hamburg, D-22607 Germany; 8 Institut fur Festkorper und Materialphysik, Technische Universitat Dresden, Dresden, Germany; 9 Helmholtz-Zentrum Berlin für Materialien und Energie, Hahn-Meitner-Platz 1, 14109 Berlin, Germany; 10 Laboratoire CRISMAT, ENSICAEN UMR6508, 6 Bd Maréchal Juin, Cedex 4, Caen, 14050 France

**Keywords:** Synthesis of graphene, Electronic properties and devices

## Abstract

In this paper we present a facile method for the synthesis of aminated graphene derivative through simultaneous reduction and amination of graphene oxide via two-step liquid phase treatment with hydrobromic acid and ammonia solution in mild conditions. The amination degree of the obtained aminated reduced graphene oxide is of about 4 at.%, whereas C/O ratio is up to 8.8 as determined by means of X-ray photoelectron spectroscopy. The chemical reactivity of the introduced amine groups is further verified by successful test covalent bonding of the obtained aminated graphene with 3-Chlorobenzoyl chloride. The morphological features and electronic properties, namely conductivity, valence band structure and work function are studied as well, illustrating the influence of amine groups on graphene structure and physical properties. Particularly, the increase of the electrical conductivity, reduction of the work function value and tendency to form wrinkled and corrugated graphene layers are observed in the aminated graphene derivative compared to the pristine reduced graphene oxide. As obtained aminated graphene could be used for photovoltaic, biosensing and catalysis application as well as a starting material for further chemical modifications.

## Introduction

In the past years derivatization of graphene has become one of the central topics in the field of nanocarbon materials studies^1,2^. Significant efforts are being made to obtain graphene derivatives covalently modified by various functional groups, containing oxygen, nitrogen, sulfur, halogens and other elements^3,4^. As a result, the family of functionalized graphenes has grown dramatically during recent years^5^. Such excitement for the graphene functionalization is a result of wide opportunities in tailoring its physical and chemical properties which are being opened by adding certain type and number of organic moieties onto either graphene basal plane or its edges. Graphene derivatization allows to tune material electrical resistivity, luminescence properties, and optical transmittance, open and vary band gap what is of high interest for electronic, optoelectronic and electrochemical applications^6–8^. The addition of chemically reactive moieties, such as carboxyls, amides or amines^9,10^ modifies graphene reactivity, surface energy and surface chemistry, substantially improving the performance of graphene-based catalysts, gas sensors, and biosensors^11–13^. And last but not least, functionalization supports the successful dispersion of graphene in organic solvents, which the main issue in processing the graphene-based materials^14^.

Besides the most known graphene derivatives, graphene oxide (GO) and reduced graphene oxide (rGO)^15–18^, as well as fluorographene and graphane^19,20^, amino-functionalized graphene is another derivative also being extensively studied nowadays. Primary amines represent attractive functionalities that enable an easy graphene grafting through amide coupling or so-called “click” reactions. Such an approach makes possible to covalently functionalize graphene with a large variety of biomolecules, particularly DNA strands and aptamers, as well as with carboxylated forms of carbon nanotubes or fullerenes^21^. Moreover, as amine is proved to be an electron-withdrawing group the functionalization of graphene with amines modify its electronic structure, in particular, enhance conductivity and provide controllable work function engineering^22^. As a net result, aminated graphene is regarded as a promising material for various applications in photovoltaic, gas sensing and biosensing, drug delivery and composite formation^12,23–25^.

Various strategies for amine functionalization of graphene are currently used. For instance, Baraket *et al*.^11^ have demonstrated successful graphene grafting with about 9 at.% of primary amines using electron beam produced Ar/NH_3_ plasma. Zhang *et al*.^24^ have also reported the formation of amino-functionalized graphene via Hoffman rearrangement using graphene oxide as a starting material with amine content of around 4 at.%. However, the applied procedure involves several stages, requires hazardous reagents and works only on the edges of graphene flakes. The hydrothermal approach is widely used for graphene functionalization and, particularly, for nitrogen doping of graphene oxide via reaction with ammonia, melamine, etc^26–28^. However, all these reactions entail the use of autoclave operating at rather high temperatures (up to 195 °C) and, therefore, mostly provide incorporation of such nitrogen-containing heterocycles as pyrroles and pyridines, than the formation of amines. Recently, one-pot graphene oxide amination and reduction via Leuckart reaction, which involves the conversion of a carbonyl group of an aldehyde or a ketone into the amine group, was reported by Aguilar-Bolados *et al*.^29^. Although the proposed method is simple and easy operational its efficiency is noticeably limited by localization of the formed amines on the edges of GO platelets. Additionally, the as-synthesized GO commonly contains a rather small amount of carbonyls (around 2–3 at.%) and the increase of their content requires additional GO processing, for instance, via liquid-phase partial reduction^30^.

Apparently, the direct substitution of GO basal plane groups (hydroxyl and epoxide ones) by amine groups with the simultaneous restoration of the sp^2^-conjugated graphene network is the most attractive and effective way to obtain amino-functionalized graphene. This cannot be done straightforwardly in mild conditions; however, one solution is to use an additional step of GO reductive bromination. Earlier it was demonstrated that GO treatment with bromine solutions or hydrobromic acid results in graphene oxide reduction and functionalization by bromine with as high as ~5 at.% bromine concentration^5,31,32^ Considering the high reactivity of bromine moieties, especially for substitution reactions, one can further easily obtain aminated graphene by treating prepared brominated graphene with ammonia.

This paper reports for the first time a scalable and facile approach for the formation of aminated graphene (rGO-Am) through two-step GO treatment with hydrobromic acid and ammonia solution in mild conditions. The effect of bromine and amine functionalization on morphology and electronic characteristics of graphene is discussed as well, providing further insights into the tuning of graphene physical and chemical properties via its derivatization.

## Results and Discussion

### Chemical composition analysis

**Figure 1:**
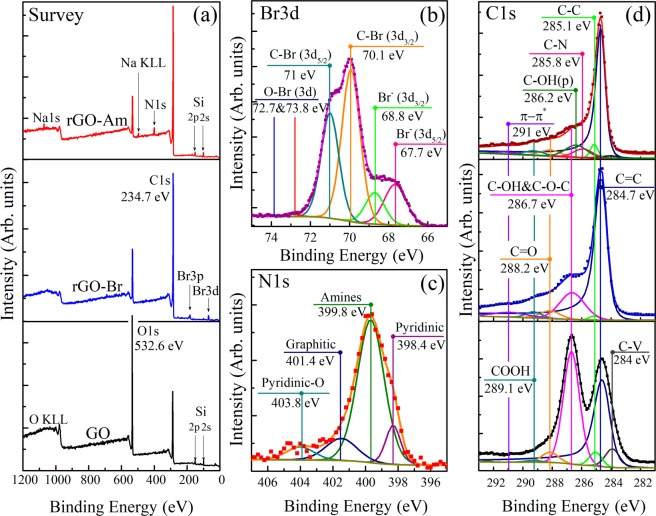
(**a**) X-ray photoelectron survey spectra of the initial GO, rGO-Br and rGO-Am. (**b**) High-resolution Br3d XPS spectrum of rGO-Br. (**c**) High-resolution N1s XPS of rGO-Am. (**d**) High-resolution C1s spectra of the initial GO and modified rGOs. For clarity, C1s spectrum and their fits are vertically offset from the fitting components. The C1s spectra were fitted by Shirley background and a set of one asymmetric Doniach-Sunjic function (DS) and five symmetric Gaussian−Lorentzian product functions (Gaussian by 70% and Lorentzian by 30%) (GL(30)), while the O1s spectra were fitted by only the GL(30) functions whose number varied from 3 to 5.

X-ray photoelectron spectroscopy (XPS) was used to determine the chemical composition of the initial graphene oxide (GO), brominated graphene (rGO-Br) and aminated graphene (rGO-Am). Figure 1a presents the survey XPS spectra. The survey spectra of the initial GO contain only C1s and O1s peaks, at ~284.7 eV and ~532.6 eV, respectively, indicating the absence of any impurities. The features related to Si near 100 eV (Si2p) and 151 eV (Si2) are due to the signal from Si substrate, underlying the studied sample. After the bromination procedure, Br3d peak at ~69 eV and Br3p doublet around ~182 and 189 eV appear, thereby confirming the presence of bromine moieties in rGO-Br sample. Considering the relevant atomic sensitivity factors, the atomic concentration of bromine calculated from the survey spectrum and was determined to be ~5.3 at.%. To analyze whether the revealed bromine is covalently bonded to graphene or just physisorbed to it the high-resolution Br3d spectrum was further measured and deconvoluted (Fig. 1b). The set of six peaks combined into three doublets are resolved in the obtained spectrum: doublet at 67.8 (Br3d_5/2_) and 68.8 eV (Br3d_3/2_) is related to free Br^−^ ^31,32^, two analogous doublets at 70.0 and 71.0 eV, and at 72.7 and 73.8 eV, respectively, correspond to bromine atoms covalently bonded to carbon^31–33^ and to oxygen^34^. The relative areas of these doublets are easily determined and are, ~23.8% for free Br^−^, 74.9% for C-Br, and 1.3% for O-Br. Accordingly, the concentration of C-Br species appears to be about ~3.96 at.%, what is comparable and even higher than the values obtained by other researchers^31–33^.

The subsequent rGO-Br treatment with the ammonia solution resulted in the complete elimination of Br3d and Br3p lines from the XPS survey spectrum and the expected appearance of N1s signal at ~400 eV. This confirms the successful substitution of bromine species by nitrogen functionalities which concentration in rGO-Am was determined to be around 5.5 at.%. The higher nitrogen content in comparison to bromine is probably due to additional incorporation of nitrogen occurring via reductive amination of the retained oxygen-containing groups in ammonia enviroment^35,36^. The curve fitting of the obtained high-resolution N1s spectrum (Fig. 1c) demonstrates the presence of four bands positioned at 398.4, 399.8, 401.4 and 403.8 eV and respectively corresponding to pyridines, amines, graphitic nitrogen and pyridine-N-oxide^36,37^. As seen from the spectrum, amine functionality with its peak area percentage of 72% appears to be a dominant type of nitrogen species, while the other ones, pyridine, graphitic nitrogen or pyridine-N-oxide, demonstrate much less relative content, not exceeding 10%. The data of NK edge XAS technique also confirms, although only qualitatively, the presence of significant amounts of amines and pyridines in the as-obtained material (Supplementary Fig. S1). The successful amination is additionally indicated by the means of Fourier-transform infrared spectroscopy (FTIR) (Supplementary Fig. S2). After the GO treatment, the characteristic absorption bands at 2970–3700 cm^−1^, 1365 cm^−1^, 1220 cm^−1^ and 980 cm^−1^ related to the interlayer water and oxygen functionalities diminish^38^. At the same time, new doublet at 3422 cm^−1^ and 3306 cm^−1^ along with peaks at 1560 cm^−1^, 1260 cm^−1^ and 795 cm^−1^ corresponding to N-H stretching of primary amines, N-H bending of primary amines, C-N stretching and N-H wag, respectively^39,40^, appear and become dominant. The additional bands at 1404 cm^−1^ 2920 cm^−1^ attributed to C-H/C-H_2_ vibrations are due to the isopropyl alcohol molecules retained after the sample preparation.

Graphene oxide bromination and subsequent amination are accompanied by the elimination of oxygen-containing groups what is implied by significant diminishing of O1s peak in the rGO-Am survey spectra. This fact is further emphasized by the detailed peak-fitting analysis of the high-resolution C1s core level spectra (Fig. 1d) in which seven distinct peaks are resolved. Three peaks centered at 283.9 eV, 284.7 eV, and 285.1 eV are respectively related to the vacancy defects of graphene lattice (peak C−V)^41^, sp^2^-bonded carbons of aromatic domains (C=C) and carbon atoms with the bonds distorted due to attachment of functional groups at a neighboring atom (C-C)^9,41–43^. The C=C peak exhibits asymmetric shape due to excitonic screening in sp^2^-conjugated graphene network of aromatic domains observed not only in graphite or graphene C1s XPS spectra but as well in the same spectra of highly reduced graphene oxide obtained by its high-temperature annealing (rGO-HT) (Supplementary Fig. S3)^43,44^. The other three peaks located at 286.7, 288.2 and 289.1 eV correspond to hydroxyl and epoxide (C-OH&C-O-C), carbonyl (C=O), and carboxyl (COOH) groups, respectively^42,45^. The last resolved peak at ~290.2 eV corresponds to π − π* shakeup satellite of the peak C=C. Quantitative analysis of the deconvoluted C1s XPS spectra (Table 1) demonstrates that initial GO has a rather high degree of oxidation with C/O ratio of 1.95. After the GO bromination, the concentration of its basal plane groups significantly reduces and the overall C/O ratio rises up to 4.18. As shown by Zheng, J. *et al*.^33^, the peak at 286.2 eV clearly observed in the XPS of brominated graphene may be assigned to the C–Br bonds. Thus, the similar broad feature observed in the C1s XPS spectrum of rGO-Br at 286.6 eV might be attributed to the sum of C–Br and C-OH&C-O-C peaks, suggesting even lower oxygen-groups content. Assuming this fact and taking into account the aforementioned bromine concentration of 3.4 at.% we obtain that the recalculated C/O ratio becomes equal to 5.02.

**Table 1:** The C/O ratios and relative concentrations of functional groups determined by deconvolution of C1s XPS spectra for the initial GO, rGO-Br and rGO-Am.

Component	Defects	C=C	C-C	C-OH & C-O-C	>C=O	O=C-OH	π-π*	C/O Ratio
Binding Energy (eV)	283.9	284.7	285.1	286.7	288.2	289.1	290.2	
GO	0.039	0.407	0.041	0.466	0.040	0.007	<0.001	1.95
rGO_Br	<0.001	0.709	0.021	0.173	0.040	0.026	0.031	4.18
rGO_Am	<0.001	0.793	0.033	0.062	0.039	0.012	0.042	8.85

After the amination step, this ratio demonstrates further growth reaching for the rGO-Am the value of 8.85, due to the additional elimination of oxygen-containing groups and by their substitution with amines. The noted value of the C/O ratio is close to that obtained for rGOs prepared by chemical reduction using common reducing agents, namely, hydrazine, benzylamine, various alcohols and sodium borohydride^18,46^. The high reduction degree of rGO-Am is also emphasized by the results of UV-Vis spectroscopy (Supplementary Fig. S4), demonstrating restoration of graphene conjugated structure. The accurate deconvolution of the rGO-Am C 1 s spectrum also revealed the appearance of the C-N peak centered at 285.8 eV^29,47^, which overlaps with the peak near 286.2 eV, corresponding to the phenol groups (C-OH(p)). These oxygen species are known to be highly stable to elimination via various reduction techniques^9,30^ retaining even after GO thermal reduction with the presence of the peaks located at 286.1–286.3 eV and 533.4–533.6 eV in the C 1 s and O 1 s XPS spectra, respectively.

### Study of the morphological features

**Figure 2:**
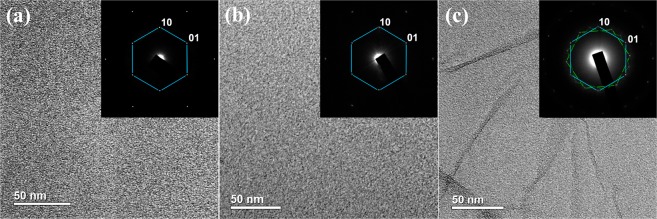
TEM images and corresponding selective area electron diffraction (SAED) patterns of (**a**) the initial GO, (**b**) rGO-Br, (**c**) rGO-Am.

The structural features of the obtained brominated and aminated graphenes were further studied by the means of different techniques such as atomic force microscopy (AFM), X-ray diffraction (XRD), transmission electron microscopy (TEM), scanning electron microscopy (SEM) and Raman spectroscopy. Representative bright field low magnification TEM images (Fig. 2) demonstrate the morphology of the initial GO, rGO-Br, and rGO-Am. No rips or nanoscale holes are observed in the initial GO, indicating its defect-free structure on the nanoscale level (Fig. 2a). The corresponding hexagonal ED patterns formed by the set of sharp spots confirmed the monolayer nature of the studied GO since characteristic intensity ratio of different spot groups, what is further verified by AFM images and XRD patterns (Supplementary Fig. S5). After the bromination procedure rGO-Br continues to exhibit lamellar defect-free structure and monolayer platelets can be distinguished in the sample (Fig. 2b). Well-preserved crystalline structure with the long-range order up to tens of nanometers retains after amination as well. However, rGO-Am demonstrates a tendency to scroll and wrinkle of initially flat graphene monolayer platelets leading to the formation of local multilayer areas distributed within single rGO-Am platelet. This is evident from the TEM image (Fig. 2c) and the corresponding ED pattern. ED pattern consists of distinguishable hexagonal diffraction patterns rotated with respect to each other (Fig. 2c). Diffraction spot intensities corresponding to the adjacent sheet areas in these diffraction patterns significantly differ from each other because of the different surface areas falling into the selective aperture of the microscope. At the same time, the intensities would be almost identical in the case of a bi- or trilayer sheets because one sheet lying under another has the same area within the aperture.

**Figure 3:**
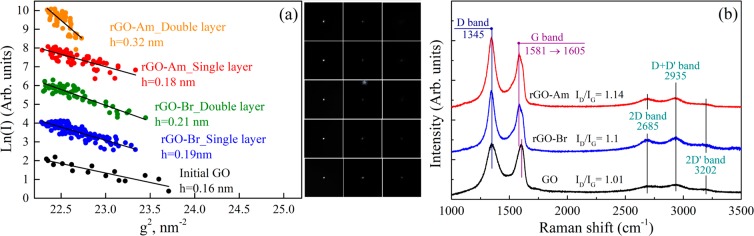
(**a**) (100) diffraction spot intensity logarithm versus reciprocal space applicate. The corresponding slopes are related to the average square of the sheet corrugation amplitude. The plots were vertically offset for clarity. Inset – the corresponding electron diffraction refluxes. (**b**) Raman spectra of the GO, rGO-Br and rGO-Am samples recorded using a 532-nm laser.

More comprehensive information on the morphology of the initial GO and functionalized graphenes was obtained by the means of the new approach developed by Kirilenko *et al*.^48^ based on the analysis of electron diffraction tilt series to determine the graphene nanorelief. The slope of the diffraction spot intensity dependence on the reciprocal space applicate square variation measured as g^2^ corresponds to the average square of the graphene sheet corrugation amplitude (more details are in Supporting Information and at the reference). Applying this method, we have found that functionalization leads to some increase of the corrugation amplitude (Fig. 3a) that results from local structural distortions caused by the bonded species. As seen from this figure, even though the concentration of functional groups on the basal plane in the case of rGO-Br and rGO-Am is substantially lower than that in the initial GO, out-of-plane distortion of the graphene layer in the modified graphenes is even higher (0.19 nm and 0.18 for brominated and aminated graphenes, respectively, which slightly exceed 0.16 nm value for the initial GO). This fact can be explained in terms of compensation of graphene net bonds distortion in GO by the opposite orientation of adjacent hydroxyl and epoxide groups with respect to the graphene net^49^. At the same time in rGO-Br and rGO-Am bromine and amine moieties are located separately and thus result in significant out of plane dislocation of carbon atoms and corrugation of graphene net.

The case of double-layered structures appears to be of even more interest. In general, when graphene and graphene oxide layers are stacked with each other they become flatter and the measured average corrugation amplitude significantly decreases^50,51^. In opposite, the studied functionalized graphenes show different behavior. The stacking of the sheets is obstructed by the functional groups what, in turn, leads to the formation of multiple knolls. As seen in Fig. 3a, in rGO-Br these knolls have the height of 0.21 nm, whereas in rGO-Am this value is 1.5 times higher and is determined to be 0.32 nm. Seemingly, knoll height should be defined by a molecular size of a functional group. However, C-Br and C-NH_2_ molecular sizes are almost the same, 0.194 and 0.197 nm respectively. We assume that higher knoll height is related to stronger electrostatic repulsion between the amine group and second graphene layer, resulting in its stronger bending and larger knoll height.

Figure 3b presents the Raman spectra of the initial GO and modified graphenes. Two major bands are commonly observed in graphene-related materials: G band at 1580 cm^−1^ originating from the in-plane stretching of the graphene lattice and D band at 1345 cm^−1^ caused by lattice disorder, particularly distortion of carbon bonds and corrugation of graphene net, as well as GO-rGO platelets edges^52,53^. In the GO Raman spectrum G band is broadened and shifted from 1580 cm^−1^ to 1605 cm^−1^ due to oxidation of the graphene net. At the same time, in the case of rGO-Br and rGO-Am two peaks at 1580 cm^−1^ and 1605 cm^−1^ are simultaneously presented in the Raman spectra, indicating restoration of sp^2^-conjugated graphene network along with the presence of localized areas functionalized with bromine or amine moieties. Functionalization also results in a slight rise of D band intensity, with the increase of I_D_/I_G_ relation from ~1 for the initial GO to 1.12 and 1.17 for the rGO-Br and rGO-Am, respectively. Considering both the aforementioned absence of observable defects and results of the electron diffraction studies the indicated rise of the D band is likely to originate from the distortion of the graphene network due to the provided covalent grafting. Both rGO-Am and rGO-Br also present three second order bands of medium intensity: 2D band (2685 cm^−1^), D + D′ band (2935 cm^−1^) and 2D′ band (3202 cm^−1^). The appearance of these bands is related to the interaction of the incorporated bromine and amine moieties in the double resonant processes that involve two phonons and was observed earlier for the aminated graphene^24^.

**Figure 4:**
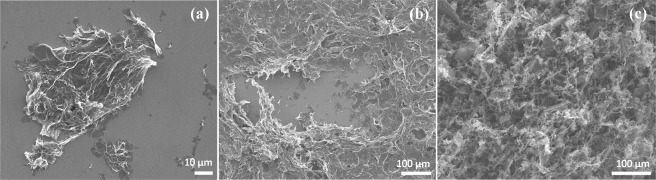
SEM images of rGO-Am (**a**) individual platelets, (**b**) multilayer film deposited on the Si wafer and (**c**) aerogel obtained by lyophilization of rGO-Am dioxane suspension.

The morphology of the obtained aminated graphenes was also studied by scanning electron microscopy (SEM) at various scales. Regardless of the solvent type used during the deposition process, which was varied from the polar ones (isopropyl alcohol) to non-polar solvents (trichloromethane and tetrachloromethane), rGO-Am platelets display a wrinkled and twisted structure (Fig. 4a). This results in the reduction of the π-π* interlayer stacking and leads to the formation of films, exhibiting irregular porous network structure (Fig. 4b). The morphological features of rGO-Am films also appear in NK edge XAS spectra, in which the absence of angular dependence of π*-resonances is observed, asserting isotropic nature of the studied sample (Supplementary Fig. S6).

To further facilitate the porous network arrangement lyophilization of rGO-Am dioxane suspension was carried out, resulting in the formation of aminated graphene aerogel (Fig. 4c). The Brunauer-Emmett-Teller (BET) specific surface area of the formed aerogel determined by N_2_ adsorption experiments has been measured to be ~265 m^2^g^−1^. This value is lower than those achieved in formation of the structured GO and rGO aerogels^54^, but still sufficiently high for rGO-Am applications in the catalysis and adsorption of metallic or dye pollutants^55^.

It is worth noting that despite the rGO-Am films contain many voids, cracks, and ripples due to the corrugation of the graphene layer, SEM studies of the arrays of individual rGO-Am platelets reveal that the applied modification procedure does not lead to tearing and reduction of the lateral size of GO platelets. The obtained SEM images demonstrate (Supplementary Fig. S7) that the rGO-Am individual platelets are of the same scale as the initial GO platelets (10–40 μm) and the size distributions of these materials are almost equal.

### Chemical reactivity of the aminated graphene

The formation of primary amines via the applied procedure and their chemical reactivity were further analyzed by a chemical test based on the reaction between rGO-Am and 3-Chlorobenzoyl chloride. This organic compound carries acyl chloride functional group, -COCl, known to react readily with primary amines forming covalent bond: so-called amide coupling^56^. This reaction commonly applied for covalent modification of amine-containing materials with species and nanocarbon structures, carrying carboxyl groups (-COOH), which can be transformed to acyl chloride moieties by the treatment with thionyl chloride (SOCl_2_), phosphorus trichloride (PCl_3_), or phosphorus pentachloride (PCl_5_)^57,58^. Thus, the chosen test reaction allows to demonstrate the feasibility of rGO-Am covalent grafting with various biomolecules, dyes, functionalized nanocarbon materials, etc.

**Figure 5:**
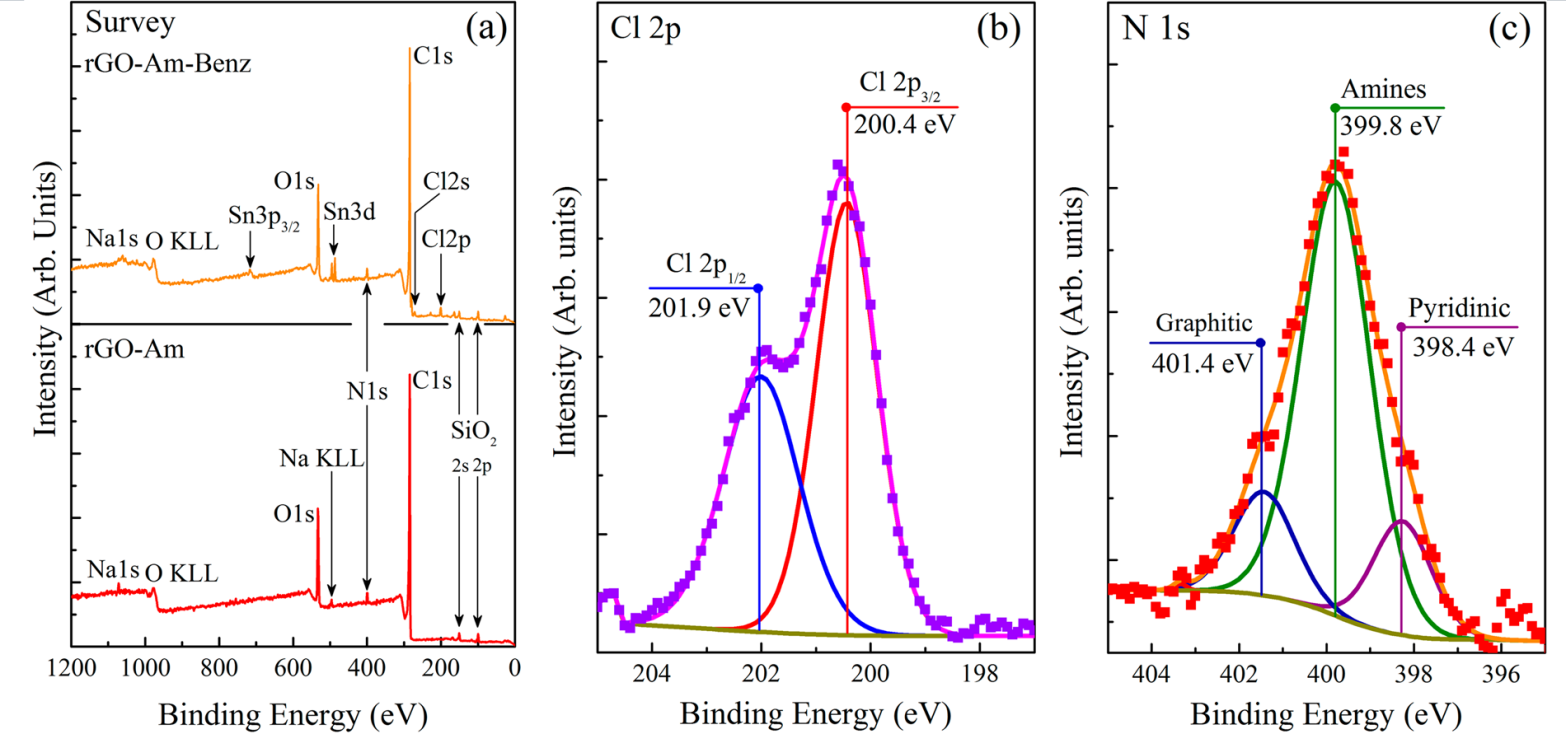
(**a**) XPS survey spectra of the rGO-Am films prior to and after test covalent modification with 3-Chlorobenzoyl chloride (rGO-Am-Benz). High-resolution (**b**) Cl2p and (**c**) N1s XPS spectra of rGO-Am-Benz sample.

To check whether the modification of rGO-Am by 3-Chlorobenzoyl chloride successfully proceeded the series of additional XPS measurements were performed. Figure 5a demonstrates the XPS survey spectra of the rGO-Am sample prior to and after the test reaction (rGO-Am-Benz). Thanks to the presence of second chlorine, which is not taking part in the amide coupling, the successful covalent bonding between the aminated graphene and 3-Chlorobenzoyl chloride is unambiguously seen from the appearing of Cl2s peak at ~272 eV and Cl2p doublet around ~200 and 202 eV. This is additionally verified by high-resolution Cl2p spectrum (Fig. 5b), where peaks at 200.4 eV (2p_3/2_) and 201.9 eV (2p_1/2_) related to the C-Cl bonds^59^ are observed. The chlorine concentration calculated from the XPS survey spectrum is ~2.8 at.%. Since as it was earlier estimated the concentration of amine groups is ~4 at.%, the efficiency of the performed amide coupling appears to be about 74% determined as a relation between the concentration of bonded chlorine and the presented amines. Besides the XPS results, rGO-Am covalent grafting by the 3-Chlorobenzoyl chloride is also indicated by the obtained FTIR spectrum (Supplementary Fig. S8), where a set of characteristic bands at 740 cm^−1^, 1170 cm^−1^, 1565 cm^−1^, 1660 cm^−1^ and 3220 cm^−1^ related to the amide bonding arise. Thus, the obtained results confirm the presence of chemically reactive primary amines. Additionally, the thermal stability of the aminated graphene was analyzed, as amines are known to easily convert in pyridines and pyrroles upon heating^36^. During the purification from the unreacted 3-Chlorobenzoyl chloride, the samples were heated up to 150 °C for 3 hours. The deconvolution and further analysis of the then-obtained N1s spectra (Fig. 5c) demonstrate that the suggested modification of rGO-Am samples does not significantly affect their chemical composition and relative concentration of the amine groups still remains to be ~72%, while the concentrations of pyridines and graphitic nitrogen appear to be about 13% and 15%, respectively.

The chemical reactivity of the rGO-Am was also verified by the test reaction of its influence on the CuCl oxidation to CuCl_2_. 200 mg of CuCl was dispersed in 20 ml of 1 M HCl and the obtained solution was divided into two parts. The first one was left stirring in the air and the second half of the solution was mixed with 50 mg of the aminated graphene and rigorously stirred for two hours. In the HCl medium CuCl is unstable and is known to transform into CuCl_2_ with the presence of Cu_2_^+^ and Cl^−^ ions in the mixture. Depending on the concentration of Cl^−^ ions the obtained solution is either yellow (Cl^−^ to Cu^2+^ is about 2:1), green (high concentration of the Cl^−^ ions) or blue (low concentration of Cl- ions). Amines from the rGO-Am are weak bases, which should interact with the Cl^−^ ions and, thus, reduce their content in the solution, leading to the blue color of the resulting solution. Indeed, this effect we observed in the case of the mixture with the addition of rGO-Am (Supplementary Fig. 9) with the retention of the CuCl + HCl solution without rGO-Am green. The observed blue color of the resulting solution can also be related to the hydrolysis of the amine groups from the rGO-Am in the aqueous medium and formation of a tetraamine copper hydroxide complex, which also induces the blue colouring of the mixture. Thus, the performed reaction additionally demonstrates the presence of chemically active amines in the obtained rGO-Am material.

### Electronic properties

**Figure 6:**
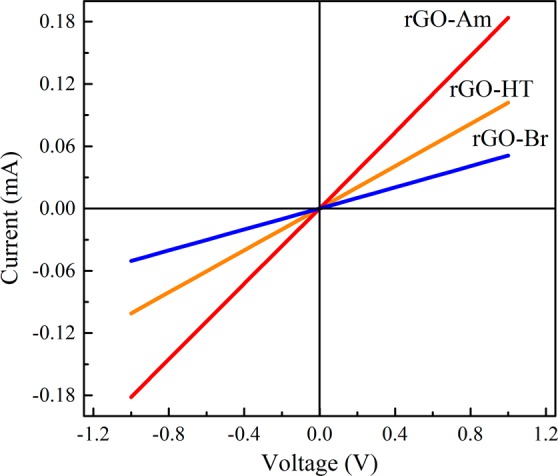
I-V curves of rGO-Br, rGO-Am and rGO-HT samples.

Besides changing of graphene chemical reactivity the presence of amines, analogously to other nitrogen species, substantially affects the electronic properties of graphene^22,60,61^. Particularly, it is expected that the rGO-Am samples due to their n-doping^61^ can demonstrate a noticeable increase in the conductivity as compared to the samples of pristine rGO with the same reduction degree (Supplementary Fig. S2). Figure 6 presents the voltage (V) versus current (I) characteristics plot of rGO-Br, rGO-Am and rGO-HT films. The V vs. I data show a linear behavior for all the samples, confirming the good Ohmic contact between the film and electrodes. The sheet resistance and electrical conductivity calculated considering the films geometry and averaged over several measurements are summarized in Table 2. As seen, rGO-Am shows 2 times higher conductivity than that in pristine rGO-HT, 270 S/m and 134 S/m, respectively. This confirms the N-doping effect from the amine groups. At the same time, rGO-Br conductivity is almost 4 times lower due to a lower degree of graphene basal plane reduction.

**Table 2:** Sheet resistance and the corresponding conductivity values of the rGO, brominated rGO and aminated rGO.

Sample	Sheet resistance, Ω/sq	Conductivity, S*m^−1^
GO	>10^12^	—
rGO_HT	4.3*10^3^	134
rGO_Br	1.1*10^4^	73
rGO_Am	2.1*10^3^	271

**Figure 7:**
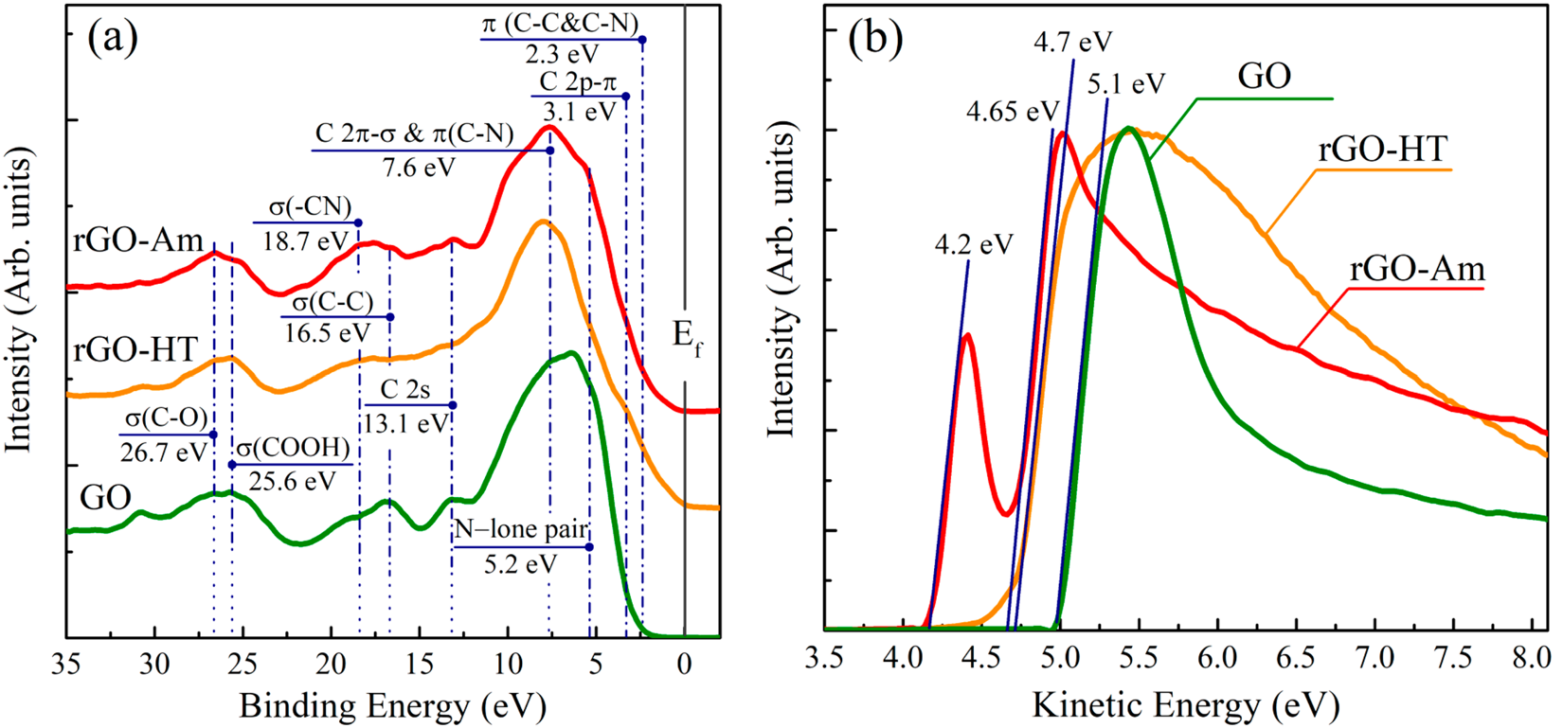
(**a**) Valence band spectra and (**b**) secondary electron energy cutoff spectra of the initial GO, rGO-Am and rGO-HT. E_f_ line corresponds to the position of Fermi level. The Fermi level position is referred at binding energy 0 eV. All the spectra were aligned considering the energy shift due to charging effect.

To explore the effect of the functionalization with amine groups on the electronic structure of graphene, valence band spectra of the initial GO, rGO-HT, and rGO-Am using a photon at 130 eV were measured (Fig. 7). All the spectra are dominated by a broad band centered at ∼7.6 eV commonly attributed to the 2p-σ electron states of graphene net^62^. Additionally, broad features at 26–28 eV, corresponding to σ electronic states in carboxyls and carbonyls are presented, indicating their presence in all samples what coincides with XPS data. At the same time, significant differences are observed in the range 0–6 eV. In the initial GO spectrum noticeable peak at ~5.5 eV corresponding to 2p π-σ overlap states, related to C-O bonds formation^63^. This assignment is justified by the observed diminishing of this feature in the valence band spectra of both rGO-Am and rGO-HT, where most part of the oxygen-containing functional groups are eliminated. Furthermore, close to zero density of states (DOS) is observed between Fermi level and ~2.2 eV in the case of GO, indicating the presence of band gap of ~4.5 eV which is in a good agreement with the published data^64^. On the contrary, both rGO-HT and rGO-Am demonstrate non-zero DOS in the region from 3 to 0 eV as a result of the increase of C2p-π electron content due to enlargement of the π -conjugated polyaromatic sp^2^-domains during the GO reduction. In rGO-HT VB spectrum peak at ~3.2 eV corresponding to C 2p-π states is more distinguishable compared to the one in rGO-Am suggesting the more complete restoration of the delocalized π-conjugated system. Note, that this difference can be also attributed to the corrugated nature of rGO-Am lattice which distortion also influences the number of 2p-π electronic states. Despite this similarity between rGO-Am and rGO-HT valence band spectra, several bands related to the introduction of the nitrogen species appear in the former one. Particularly, the bands at ~5.0 eV and ~7.3 eV, corresponding to electronic states of N lone pair and the delocalized C–N π bonds can be distinguished in the rGO-Am valence band spectra^65,66^. Moreover, slight changes in the density of states near 2.3 eV arise due to the states related to C–C and C–N π bonds, existing in a planar sp^2^-graphene structure^67^. Finally, a broad peak centered between 18 and 20 eV can be observed after amination. While studying N-doped HOPG Favaro *et al*.^68^ have discovered the appearance of such a feature in the VB spectra of HOPG upon its N implantation and assumed that it originates from the presence of the –CN groups. Considering the absence of this peak in the spectrum of the rGO-HT sample and following Favaro *et al*. we assume that it is related to the electronic states arising due to the incorporated amines.

The effect of the amine functionalization on the graphene work function (WF) has also been studied (Fig. 7). The work functions of GO, rGO-HT and rGO-Am samples were determined using a standard approach based on subsequent measuring of the valence band and corresponding secondary electrons (SE) cut-off spectra (Fig. 7a,b, respectively). In this case, the value of work function, usually denoted as eΦ_m_, can be calculated using the following equation: eΦ_m_ = hν – (E_F_ – E_SEC_), where hν = 130 eV is the photons energy, E_F_ and E_SEC_ are the positions of Fermi level and cut-off threshold both represented in the kinetic energy scale^69^. The obtained in such a way eΦ_m_ values are represented in the Fig. 7b and equal respectively to ~4.2 eV for rGO-Am, to ~4.7 for rGO-HT and to ~5.1 eV for initial GO. It is worth noting that the value of the rGO-HT work function (~4.7 eV) is in a very good consistency with the literature data^70,71^. On the contrary, the value of the work function of initial GO (~5.1 eV) appears to be somewhat higher the typical values (~4.9 eV) obtained by other groups^71,72^, probably due to the higher degree of graphene oxidation in our case.

Interestingly, the SE spectrum of rGO-Am demonstrates the presence of two slopes indicating that the surface of this sample consists of two types of domains. The work function of first type domains has the value of ~4.2 eV, while for second type domains it becomes very close to the work function of the rGO-HT sample (~4.65 eV). This fact points out the substantial local decrease of the rGO-Am work function reaching the value of ~0.5 eV (see Fig. 7b). The primary amines located along the edges and wrinkles of graphene platelets are the electron-donating moieties, and, as that, have to cause some decrease in the rGO work function^72,73^. Hence, the observed decrease in rGO-Am work function may be attributed to the contribution of the amines as well as pyridines, which both form a noticeable amount of rGO domains with the lower value of eФ_m_.

## Conclusion

In summary, the approach for scalable production of the aminated graphene from graphene oxide is developed. The proposed method leads to the reduction of graphene and subsequent incorporation of up to 4 at.% of amines with low content of other nitrogen species (pyridines and graphitic N) as indicated by XPS and XAS data. Note, that the amination efficiency can be further enhanced by increasing Br concentration via modification of the bromination method. Both TEM and SEM studies revealed that due to the presence of amines and elimination of oxygen functional groups, the aminated graphene exhibits complicated morphology with a tendency to form wrinkled and corrugated structure. This facilitates the formation of the porous films and aerogels from the obtained aminated graphene, making it perspective for electrocatalyst and sensing applications. The use of the aminated graphene in biosensing applications, particularly aptasensors manufacturing, is further enhanced by its chemical reactivity through amide coupling what is emphasized by the successful covalent linking of 3-Chlorobenzoyl chloride to the obtained material. In addition, the amination increases the conductivity of graphene layers and alters its valence band structure and work function that is of interest not only for optoelectronic and photovoltaic applications but also for the study properties of variously functionalized graphenes.

## Methods

### GO synthesis and chemical modification

Graphene oxide was synthesized by the Hummers method^74^. Graphite powder (4 g) was oxidized by using concentrated H_2_SO_4_, KMnO_4_ and 30% H_2_O_2_ solution. No nitrates were used to prevent nitrogen doping of graphene oxide during the synthesis. The rest of the GO preparation procedure is analogous to that described in our previous work^30^ and its main steps are as follows. The resulting mixture was centrifuged at 3500 rpm for 1 hour, and the supernatant was decanted away. The remaining material was additionally centrifuged at 1500 rpm for 10 min to obtain aqueous GO suspension as a supernatant. Sonication was excluded throughout the whole process to prevent damaging of graphene oxide flakes and obtain suspensions with GO flakes lateral size up to 100 μm.

Graphene oxide bromination was performed as follows. The GO aqueous suspension (0.05 wt.%) was centrifugated (18186 g, 15 minutes), the supernatant was decanted away. After that, HBr acid (46%, Sigma-Aldrich) was added to the sediment and suspension was intensively shacked for 60 seconds. The described procedure was repeated three times and the finally obtained suspension was stirred using magnetic stirrer during 20 hours in closed flask. The obtained brominated graphene was copiously washed with centrifugation (18186 g, 25 minutes) and rinsing the obtained sediment with organic solvent by mechanical stirrer (5 minutes stirring). This procedure was repeated 5 times. During the first 2 cycles chloroform was used as a solvent and in the last 3 cycles it was changed to the isopropyl alcohol.

Amination was carried out by centrifugation (18186 g, 15 minutes) of rGO-Br isopropyl alcohol suspension (0.05 wt.%), decanting the supernatant away and rinsing the sediment by a saturated solution of ammonia in isopropyl alcohol. The described procedure was repeated 3 times. The washing procedure is the same as in the case of rGO-Br synthesis.

Covalent modification of rGO-Am by 3-Chlorobenzoyl chloride was performed as follows. rGO-Am was filtered using a glass filter and 3 times washed with acetonitrile. Afterwards, 50 mg of rGO-Am was dispersed in 10 ml of acetonitrile and 100 μl of triethanolamine and 50 μl of 3-Chlorobenzoyl chloride were added while stirring. The obtained suspension was further stirred for one hour in a closed flask. The as-prepared suspension was filtered using glass filter with subsequent washing using acetonitrile, deionized water, ethyl alcohol, and chloroform.

### Characterization of the initial GO and the obtained modified graphenes

A set of characterization techniques was exploited similarly to the used in the aforementioned work^30^ with some extensions concerning the study of the aminated and brominated materials as follows. Measurements using X-ray photoelectron spectroscopy (XPS) were made using Thermo Fisher ESCALAB 250Xi XPS system equipped with an Al Kα X-ray source providing 1486.6 eV line. Calibration of the spectra was performed using the Au 4f7/2 line at 84.0 eV as a reference. Effect of surface charging of low-conducting GO surface was treated by aligning of XPS spectra according to the C1s line at 284.6 eV of a conductive rGO-HT (see Supplementary Fig. S2). CasaXPS software was used for quantification and fitting of the XPS spectra. Nonlinear least-squares routine was used for the χ2 minimization. Shimadzu-2450 spectrophotometer was used for the UV-vis absorption spectra acquisition from the studied samples. Fourier transform infrared spectroscopy was performed on the Infralum-08 FTIR spectrometer equipped with the attenuation of total reflectance attachment. Horiba Jobin-Yvon LabRam HR800 apparatus equipped with a Laser Quantum Torus 532-nm laser having 50 mW of the output power was used for Raman spectroscopy. The excitation source was attenuated with an optical density 1 filter condensed by a 20x lens to a 30 µm spot. The light power at the sample was 0.11 mW. The set of five Raman spectra were obtained in different spots of the studied samples and further averaged to provide reliable data. Measurements of electrical conductivity in the GO, rGO-Br and rGO-Am samples were performed using a two-electrode system. Thin films of the studied material were obtained by casting a drop of the corresponding suspension onto quartz substrates with two comb 80 nm thick Au electrodes with 500 µm separation prepared on them. The electrode comb included 8 electrode bar pairs (Supplementary Fig. S10).

N-K edge X-ray absorption spectra (XAS), Valence band spectra and Work function spectra were recorded at the Russian-German beamline of electron storage ring BESSY-II (Helmholtz-Zentrum Berlin) using the beamline ultrahigh vacuum experimental station^75^. The XAS measurements were performed in the total electron yield (TEY) mode realized by sweeping the incident photon energy and simultaneously recording the sample drain current. The thus-obtained TEY XAS spectra were then subjected to appropriate normalization and smoothing.

Structural characterization was mainly performed analogously to that presented in our previous work^30^ with some differences as can be seen in the following. X-ray diffraction (XRD) technique was implemented using Bruker Smart Apex Duo set-up equipped with a CuKα source and an Apex 2D detector. Sample for these studies was prepared by the fixation of a material portion on a cactus needle end by a nitrocellulose lacquer. Then a series of diffraction patterns were acquired at various incidence angles (of the X-ray beam incidence on the detector), and the obtained 2D data was recalculated to the means of 2θ scan. DIFFRAC.EVA (Bruker Cor.) software was used for the obtained diffraction curves analysis based on the data from Powder Diffraction File ICCD PDF-2 release [JCPDS-International Centre for Diffraction Data (http://www.icdd.com)]. The transmission electron microscopy including electron diffraction (ED) studies were performed on a JEM ARM200F cold FEG probe and image aberration corrected electron microscope equipped with a large solid-angle CENTURIO EDX detector, Gatan GIF QUANTUM, and ORIUS CCD camera and Jeol JEM-2100F microscope. TEM sample was prepared by wetting a TEM-grid with carbon lacey film in a diluted water dispersion of the studied material. Jeol JSM-7001F microscope was used for SEM studies. Langmuir−Blodgett method was exploited for monolayer films formation in a way described elsewhere^76^. AFM images were obtained using Veeco Dimension 3100 atomic force. Operation in tapping mode using RTESP probes was used for surface morphology and thickness of the rGO-Am films determination.

Specific surface area was measured by Brunauer-Emmett-Teller (BET) method. Immediately prior to the measurements, the sample of rGO-Am was preheated to 180 °C and kept at this temperature for an hour in a vacuum to remove any products that could be adsorbed on the highly developed surface of the sample.

## Supplementary information

Supplementary information.
