# Supplementary material for: From graphene oxide towards aminated graphene: facile synthesis, its structure and electronic properties
Source: Sci Rep. 2020 Apr 23;10:6902. doi: 10.1038/s41598-020-63935-3 (PMC7181732; doi:10.1038/s41598-020-63935-3)
Supplement: Supplementary file 1 — Supplementary information. [file 41598_2020_63935_MOESM1_ESM.pdf]

Supporting information

From graphene oxide towards aminated graphene: facile synthesis, its structure and electronic properties

Maxim K. Rabchinskii,[1] Sergei A. Ryzhkov,[1] Demid A. Kirilenko,[1,2]* Nikolay V. Ulin,[1] Marina V. Baidakova,[1,2] Vladimir V. Shnitov,[1] Sergei I. Pavlov,[1] Ratibor G. Chumakov,[3] Dina Yu. Stolyarova,[3] Nadezhda A. Besedina,[4] Aleksandr V. Shvidchenko,[1] Dmitry V. Potorochin,[2,6,7] Friedrich Roth,[6] Dmitry A. Smirnov,[8] Maksim V. Gudkov,[5] Maria Brzhezinskaya,[9] Oleg I. Lebedev,[10] Valery P. Melnikov[5] and Pavel N. Brunkov[1,2]

[1] Ioffe Institute, 26 Politekhnicheskaya, 194021 Saint Petersburg, Russia

[2] ITMO University, 49 Kronverksky Pr., 197101 Saint Petersburg, Russia

[3] NRC "Kurchatov Institute", 1 Akademika Kurchatova pl., 123182 Moscow, Russia

[4] St. Petersburg Academic University, Khlopin St. 8/3, 194021 Saint Petersburg, Russia

[5] Semenov Institute of Chemical Physics of Russian Academy of Sciences, Kosygina St., 4, 119991 Moscow, Russia

[6] Technische Universität Bergakademie Freiberg, Akademiestraße 6, 09599 Freiberg, Germany

[7] Deutsches Elektronen-Synchrotron DESY, 85 Notkestraße, Hamburg, D-22607, Germany

[8] Institut fur Festkorper und Materialphysik, Technische Universitat Dresden, Dresden, Germany

[9] Helmholtz-Zentrum Berlin für Materialien und Energie, Hahn-Meitner-Platz 1, 14109 Berlin, Germany

[10] Laboratoire CRISMAT, ENSICAEN UMR6508, 6 Bd Maréchal Juin, Cedex 4, Caen-14050, France

* - Corresponding author: Demid A. Kirilenko, E-mail address: Demid.Kirilenko@mail.ioffe.ru
Tel: +7 905 226 29 13 Fax: +7 812 297 00 73

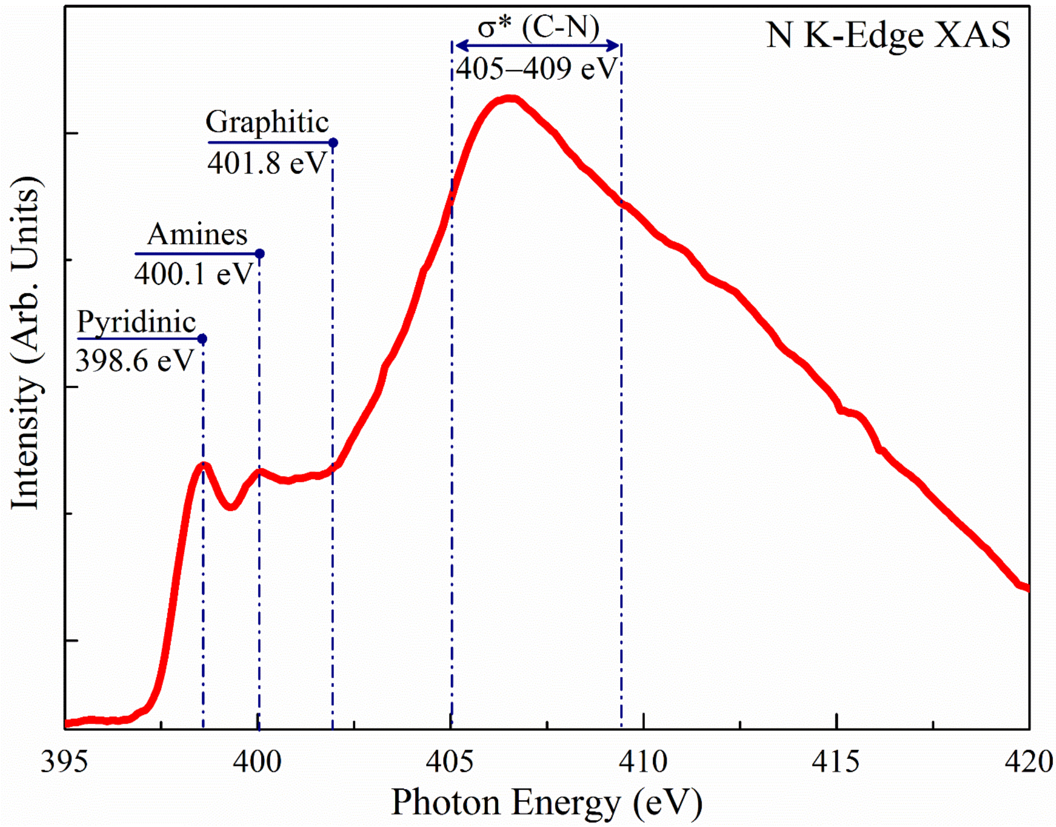

Figure S1| NK edge XAS spectrum of rGO-Am sample obtained in the total electron yield mode by recording the drain current of the rGO-Am layer deposited on silicon substrate.

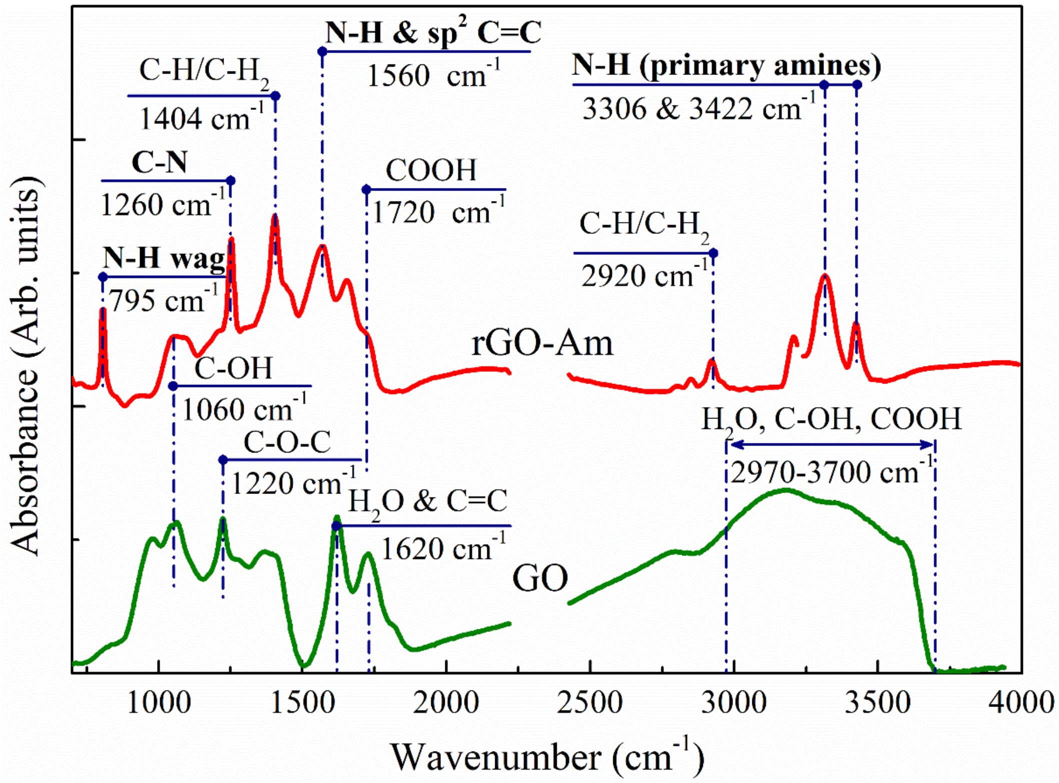

Figure S2 | FTIR spectra of the initial GO (green curve) and aminated rGO (red curve)

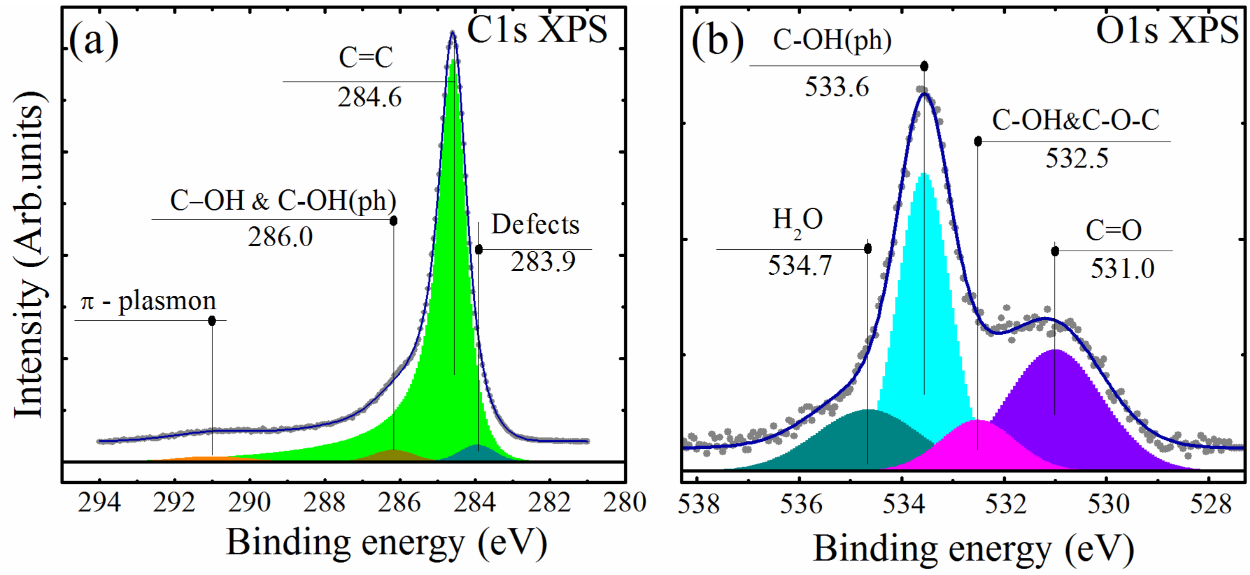

Figure S3 | High-resolution (a) C1s and (b) O1s XPS spectra of the initial graphene oxide sample reduced by annealing at 600 °C for 2 hours. The spectra were used as the references for the alignment and interpretation of all obtained in this work XPS spectra of GO and rGO samples.

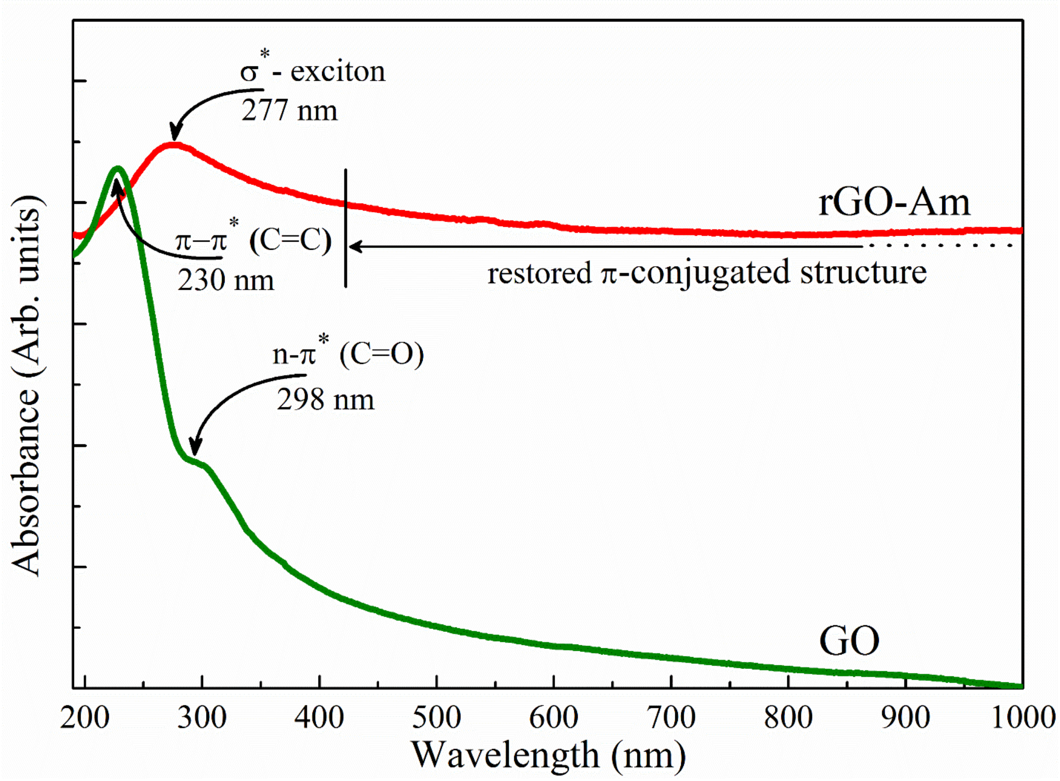

Figure S4 | UV-Vis spectra of the initial graphene oxide and obtained aminated graphene. The observed 230 nm peak redshift to higher wavelengths (~ 277 nm), vanishing of the shoulder at 300 nm disappears and substantial rise of the overall absorption in the range up to near-infrared region signifies nearly complete elimination of functional groups from the GO basal plane and restoration of sp^2-conjugated graphene network.

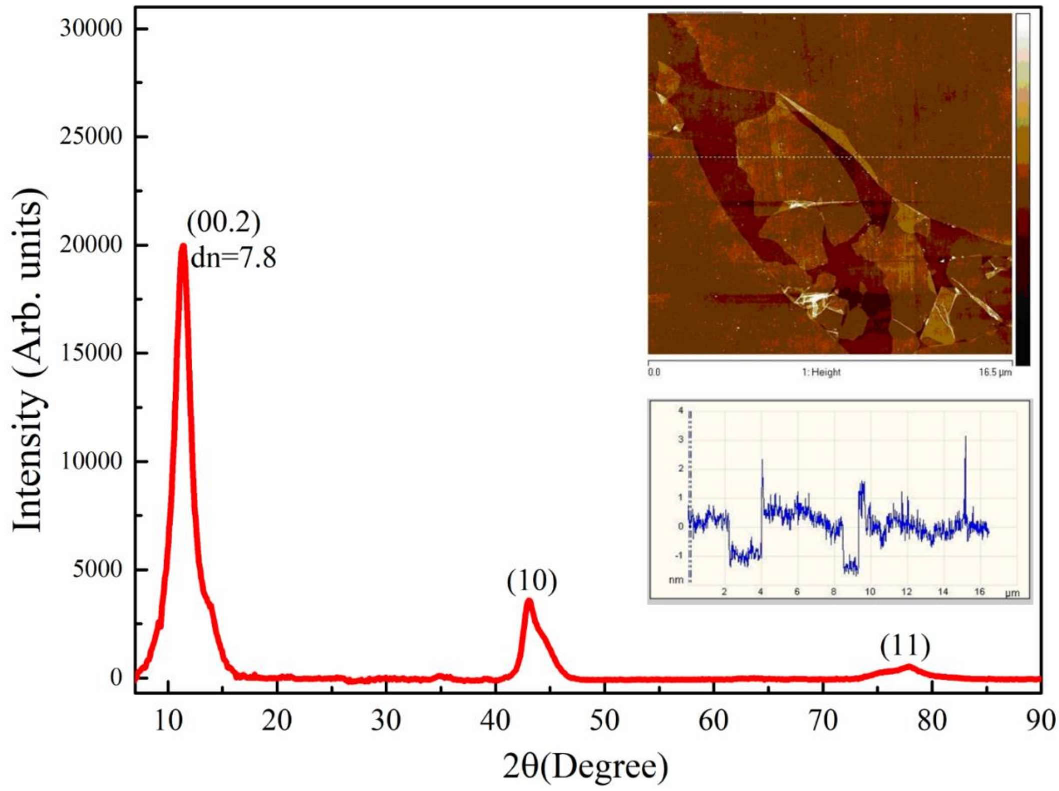

Figures S5 | XRD pattern of the initial GO. The d-value is in Å. The (10.l) and ((11.l) indicate diffraction reflections, corresponding to the superposition of crystalline reflections of type (hk.l) and two-dimensional lattice reflections of type (hk). (Inset) Atomic-force microscopy image of graphene oxide deposited on silicon wafer. The corresponding height profile is shown at the bottom.

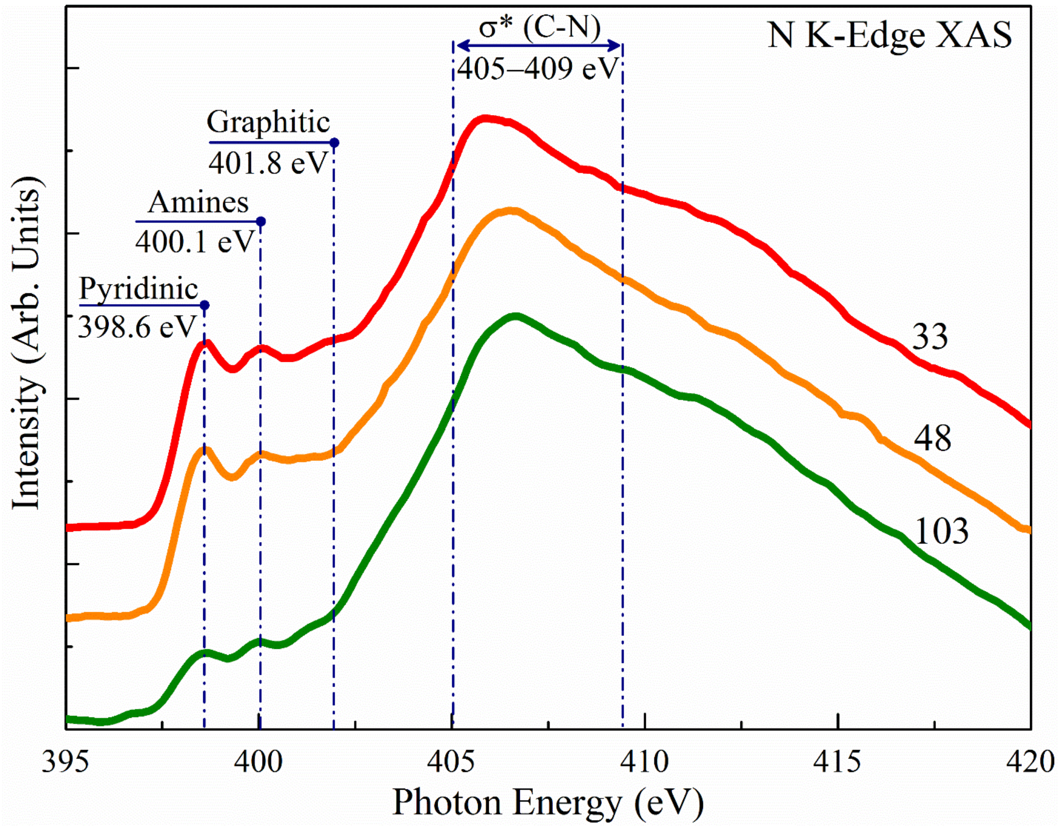

Figure S6 | NK edge XAS spectra of the aminated graphene film obtained under different angles of the radiation. All spectra were measure in the total electron yield mode by recording the drain current of the rGO-Am layer (~500 nm thick) deposited on silicon substrate.

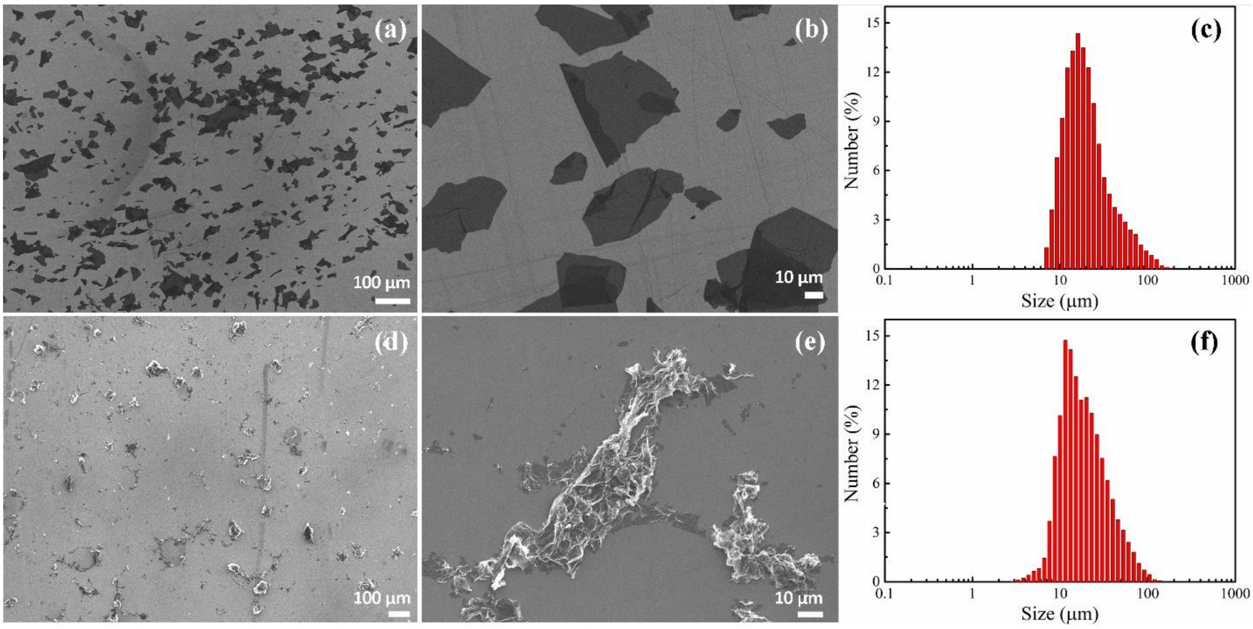

Figure S7 | (a,b) SEM images and (c) corresponding size distribution histograms of (a-c) the initial graphene oxide and (d-f) aminated graphene. The number of GO or rGO-Am platelets accounted in the distributions are 653 and 575, respectively.

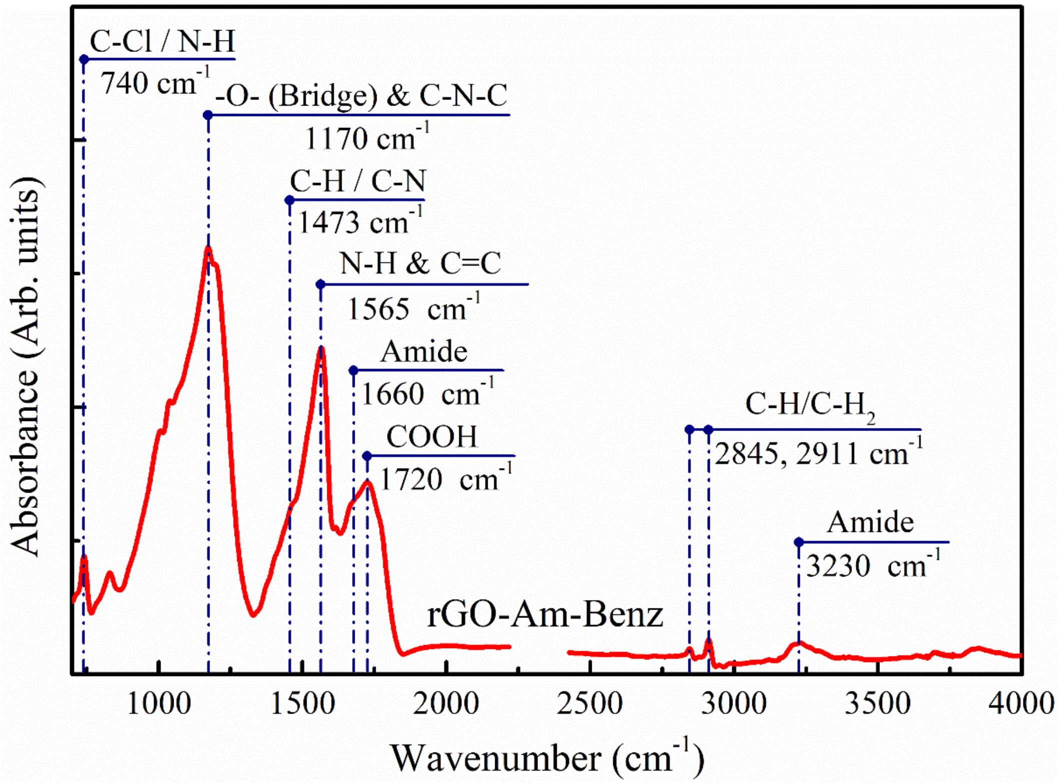

Figure S8 | FTIR spectrum of the rGO-Am-Benz sample, indicating successful amide bonding between the rGO-Am layer and 3-Chlorobenzoyl chloride

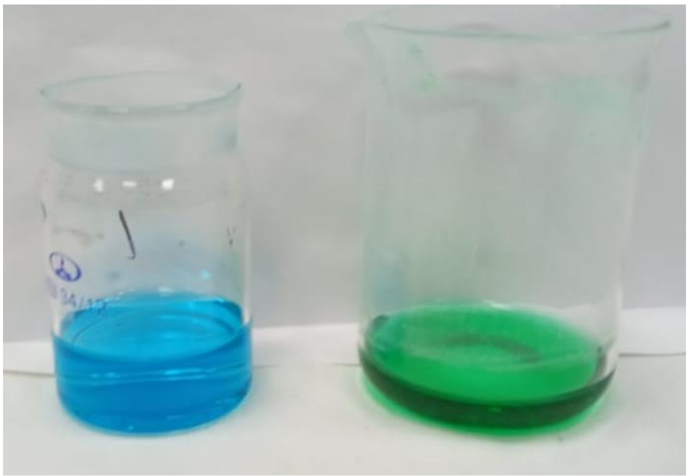

Figure S9 | The mixture of CuCl and HCl wih (left flask) and without (right flask) rGO-Am. The blue color of the solution indicates the interaction of the amines of rGO-Am with the Cl^- ions and formation of tetraamine copper hydroxide complex due to amines hydrolysis and their detachment from the rGO-Am layers.

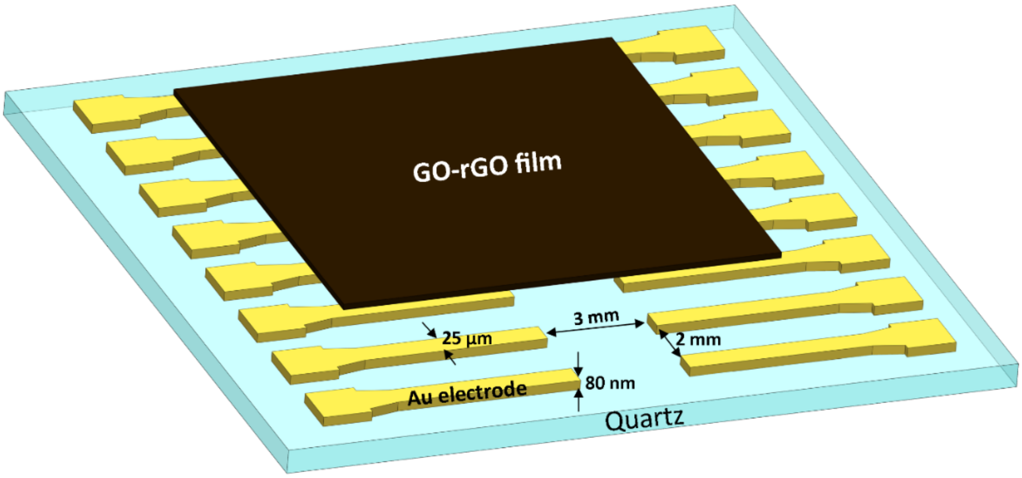

Figure S10 | The electrode substrate model for conductivity measurements of GO and rGO films. Not in scale.

Determination of the corrugation amplitude of 2D crystals by the means of electron diffraction

Corrugation amplitude of 2D crystals can be measured by electron diffraction (for example, D. Kirilenko et al. Measuring the corrugation amplitude of suspended and supported graphene. Phys. Rev. B **84**, 235417 (2011); J. Thomsen et al. Suppression of intrinsic roughness in encapsulated graphene, Phys. Rev. B **96**, 014101 (2017)). It can be done by measuring diffraction spot intensity decrease with the crystal tilt against the incident electron beam.

Electron diffraction pattern obtained in TEM represents a practically flat cross-section of the crystal reciprocal lattice which direction is defined the angle between electron beam direction and the crystal. In the case of corrugated 2D crystal reciprocal lattice consists of vertical rods and diffused clouds. Tilting crystal brings cross-sections of the reciprocal lattice nodes at higher points what is seen as corresponding change of a diffraction spot as shown in Figure S11.

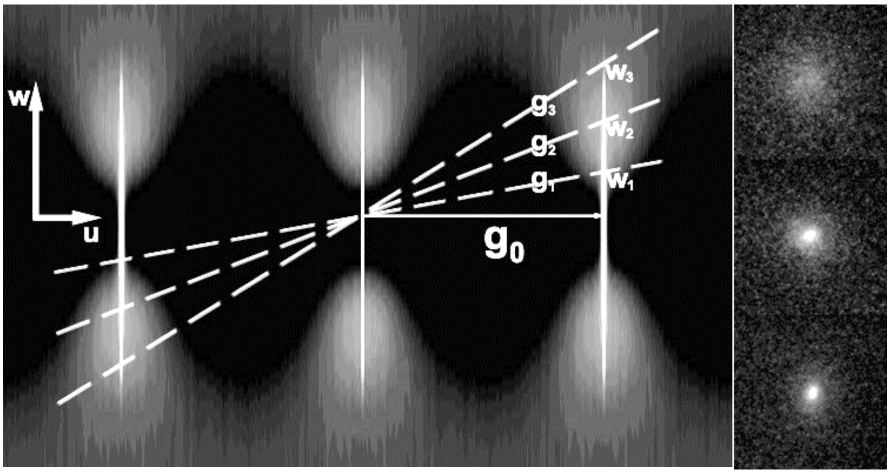

Figure S11. Side-view of reciprocal lattice of corrugated graphene with shown cross-sections representing electron diffraction patterns acquired at different tilt angles and corresponding diffraction spot evolution as it appears in experiment.

Dependence of the spot intensity and shape on the vertical coordinate *w* is described by the following formula, where *h(x,y)* – the corrugation profile:

$$F(u,v,w) = \iint_{XY} e^{2\pi i w h(x,y)} e^{2\pi i (ux+vy)} dydx \tag{1}$$

As can be derived from (1), the intensity of a diffraction spot *I* measured in its very center is described by the following:

$$I = I_0 e^{-(2\pi w)^2 \overline{h}^2} \tag{2}$$

Thus, if one measures diffraction spot intensity variation with crystal tilt we can find the average square of the corrugation amplitude $\overline{h^2}$.

The discussed technique has an important advantage in comparison to AFM. Each electron diffraction spot corresponding to the graphene structure is formed by summation of electron waves coherently scattered by graphene atoms. Thus, dependence of the spot intensity on the electron beam incidence angle represents the very information on the graphene lattice distortions. At the same time, an AFM tip would move above the functional groups and the corrugation amplitude determined by AFM includes not only graphene sheet distortions but also a contribution of the functional groups geometry itself (Figure S12).

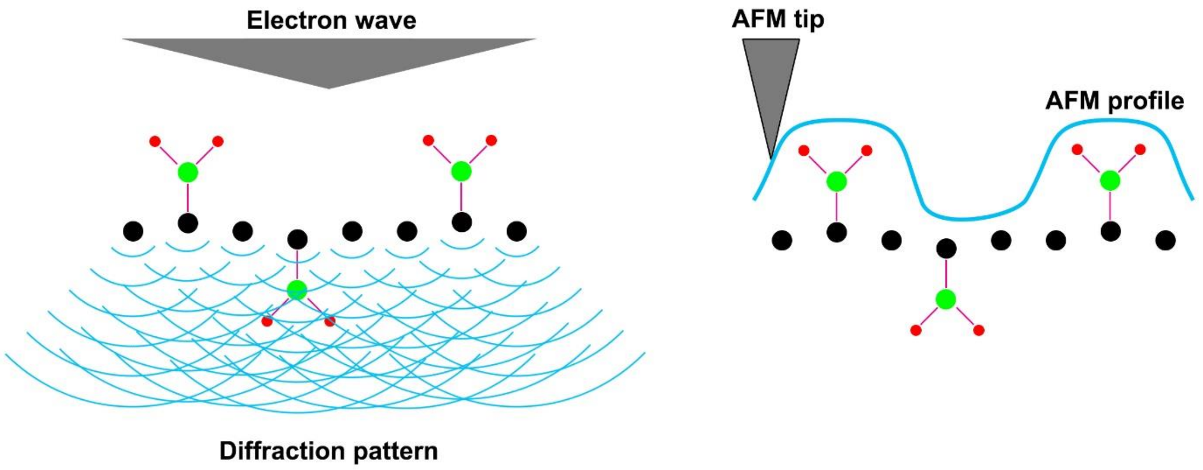

Figure S12. Schematic of differences in experimental data obtained by TEM and AFM on the corrugation of functionalized graphene.
